# Complete genome sequence of biocontrol strain *Paenibacillus peoriae* HJ-2 and further analysis of its biocontrol mechanism

**DOI:** 10.1186/s12864-022-08330-0

**Published:** 2022-02-24

**Authors:** Aiming Jiang, Chengwu Zou, Xiang Xu, Zunwei Ke, Jiangan Hou, Guihe Jiang, Chunli Fan, Jianhua Gong, Jiguang Wei

**Affiliations:** 1 College of Agriculture, Guangxi University, Nanning, 530004 China; 2 College of Chemistry and Environmental Engineering, Hanjiang Normal University, Shiyan, 442000 China; 3 Institute of Basic Medical Sciences, Hubei University of Medicine, Shiyan, 442000 China

**Keywords:** *Paenibacillus peoriae*, *Paris polyphylla*, Stem rot, Genome analysis, Biocontrol mechanism

## Abstract

**Background:**

*Paris polyphylla* is a herb widely used in traditional Chinese medicine to treat various diseases. Stem rot diseases seriously affected the yield of *P. polyphylla* in subtropical areas of China. Therefore, cost-effective, chemical-free, eco-friendly strategies to control stem rot on *P. polyphylla* are valuable and urgently needed.

**Results:**

In this paper, we reported the biocontrol efficiency of *Paenibacillus peoriae* HJ-2 and its complete genome sequence. Strain HJ-2 could serve as a potential biocontrol agent against stem rot on *P. polyphylla* in the greenhouse and field*.* The genome of HJ-2 consists of a single 6,001,192 bp chromosome with an average GC content of 45% and 5,237 predicted protein coding genes, 39 rRNAs and 108 tRNAs. The phylogenetic tree indicated that HJ-2 is most closely related to *P. peoriae* IBSD35. Functional analysis of genome revealed numerous genes/gene clusters involved in plant colonization, biofilm formation, plant growth promotion, antibiotic and resistance inducers synthesis. Moreover, metabolic pathways that potentially contribute to biocontrol mechanisms were identified*.*

**Conclusions:**

This study revealed that *P. peoriae* HJ-2 could serve as a potential BCA against stem rot on *P. polyphylla*. Based on genome analysis, the genome of HJ-2 contains more than 70 genes and 12 putative gene clusters related to secondary metabolites, which have previously been described as being involved in chemotaxis motility, biofilm formation, growth promotion, antifungal activity and resistance inducers biosynthesis. Compared with other strains, variation in the genes/gene clusters may lead to different antimicrobial spectra and biocontrol efficacies.

**Supplementary Information:**

The online version contains supplementary material available at 10.1186/s12864-022-08330-0.

## Background

*Paris polyphylla* var. *chinensis* (Franch.) Hara. is a herb widely used in traditional Chinese medicine (TCM) to treat various diseases (e.g., hemostasis, abscess, snake bite, abnormal uterine bleeding, tumors and analgesia) [1–4]. Large scale application of *Paris* in TCM helps economic value of herb increase in a dramatic way in China and other Asian countries. Yet, with the rapidly rising in demand, wild individuals of these plants have been overexploited for the last several decades. Many *Paris* (e.g., *Paris polyphylla*, *Paris fargesii* and *Paris mairei*) have been listed as endangered species in China from International Union for Conservation of Nature (IUCN). Artificial cultivation is an effective means to meet the growing demand for Chinese herbal medicine. The cultivated area of *P. polyphylla* in Yunnan had exceeded 1333 hm^2^ at the end of 2014. However, severity soilborne diseases (e.g., Stem rot, Anthracnose and Gray mold) seriously affected the yield of *P. polyphylla* [5–8]. Stem rot on *P. polyphylla*, caused by two species of *Fusarium*, *Fusarium concentricum* and *Fusarium oxysporum* is prevalent in subtropical areas of China where plants grow under rainfed conditions [7, 8]. Plants with stem rot disease developed stem cracking, shriveling, yellowing, stunting, and finally wilting, and symptoms of plant death may eventually appear within a few weeks [9, 10]. Stem rot on *P. polyphylla* ultimately limited the growth of roots as primary medicinal parts and amount of seeds. The economic control approaches of stem rot on *P. polyphylla* are challenging due to the long-term survival of mycelia in soil, weather conditions and the evolution of new races. Current management options for this disease are mainly dependent on the use of chemical management measures [11]. Extensive applications of commercially fungicides contribute to resistance in fungal pathogens. Moreover, chemical pesticides and fungicides are forbidden to use in the planting process of Chinese herb in light of health issues. Therefore, cost-effective, chemical-free, eco-friendly strategies to control stem rot on *P. polyphylla* are valuable and urgently needed.

Biocontrol has been considered a viable alternative method due to the advantages of environmental friendliness, safety and the lack of the induction of pesticide resistance [12]. The microorganisms, most of which are *Bacillus*, *Pseudomonas* and *Paenibacillus* spp., have been successfully applied for suppressing soil-borne pathogens [13–16]. Researches on the biocontrol of stem rot are still in progress and revealing new strategies. Plant growth-promoting rhizobacteria (PGPR) produces phytohormones such as cytokinins, gibberellins, indole-3-acetic acid (IAA), and protects plants against pathogens through antibiotic biosynthesis. Meanwhile, PGPR exhibits the abilities of nitrogen fixation, phosphate solubilization, siderophore production [17, 18]. In addition to these effects, many PGPRs increase plant resistance to pathogen via the elicitation of induced systemic resistance (ISR), which is triggered by a range of secondary metabolites referred to as ‘elicitors’ [19, 20]. Different signaling pathways, such as the jasmonic acid (JA) and ethylene (ET) pathways, are activated to induce plant resistance [21–23]. Although a large number of microbe species that could serve as biocontrol agents (BCAs) to manage plant pathogens have been discovered, researches on the biocontrol of stem rot on *Paris* are scarce.

In this study, we identified an efficient biocontrol strain, *Paenibacillus peoriae* HJ-2, which was isolated from the rhizosphere of *P. polyphylla*. The results of greenhouse and field experiments indicated that *P. peoriae* HJ-2 could serve as a potential BCA against stem rot on *P. polyphylla*. Whole-genome sequencing of PGPRs facilitates studies of gene mutation and molecular evolution mechanisms. Chen (2007) revealed the resistance mechanisms of *Bacillus amyloliquefaciens* FZB42 toward phytopathogen via producing antifungal components by genome analysis [24]. According to gene function annotation, signaling pathways of volatile compounds emitted from *B. amyloliquefaciens* FZB42 were described in detail. Andrés-Barrao (2017) analyzed *Enterobacter sp*. SA187 genome and revealed its plant growth promotion mechanisms for *Arabidopsis thaliana* under salt stress [25]. The genome of *Paenibacillus polymyxa* HY96-2 was sequenced, and the variation in secondary metabolites genes or gene clusters could result in different antimicrobial activities and biocontrol efficacies between HY96-2 and other *p. polymyxa* strains [26]. Furthermore, although *P. peoriae* is a potential BCA, there are few studies about biocontrol mechanism of *P. peoriae* using genome analysis or other molecular methods so far. Moreover, the differences in the biocontrol mechanisms could be revealed on the basis of comparison of genes/gene clusters. To understand the molecular mechanism involved in plant–microbe interactions, we provide a high quality genome assembly and annotation of *P. peoriae*HJ-2.

Thus, the aims of this study were to (1) identify the antagonistic activity of *P. peoriae* HJ-2 against *Fusarium* spp*.* in vivo, (2) evaluate plant growth promotion and biocontrol efficiency of *P. peoriae* HJ-2 in the greenhouse and field, and (3) compare the genes/gene clusters involved in biofilm formation, antibiotic and resistance inducers synthesis with other *P. peoriae* strains.

## Results

### Genomic characterisation of strain HJ-2

**Fig. 1:**
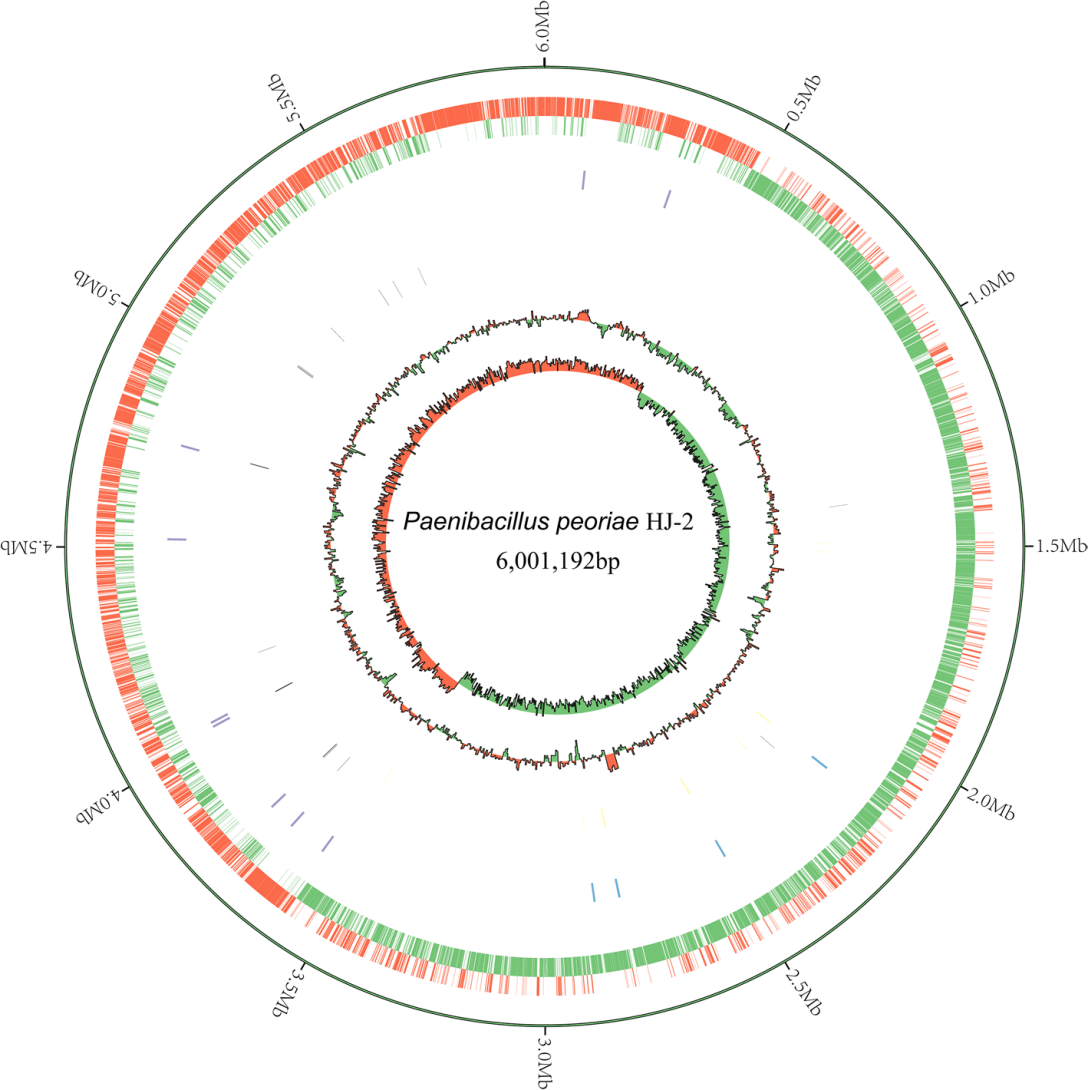
Genome map of *P. peoriae* HJ-2. The bacterial chromosome is 6.0 Mb in size. The distribution of the circle from the outside indicates the genome size, forward CDS, reverse CDS, repeat sequence, tRNA(black), rRNA(blue), GC ratio(red and green indicate regions where the GC ratio is higher than average and lower than average, respectively), and CG skew positive (red) and negative (green)

The complete genome of HJ-2 consists of a single circular chromosome of 6,001,192 bp with an average GC content of 45% (Fig. 1). Genomic DNA sequencing generated 180,325 reads and contained 1,291,048,950 bp, and the sequencing coverage reached 215 × . In total, 5439 genes were identified, including 5237 coding sequences genes (CDSs), 39 rRNA and 108 tRNA genes. The general features are shown in Table 1. Ten putative GIs were found in HJ-2 using the GI prediction methods, and the size of GIs ranged from 9.8 to 35 kb. CRISPRs contain multiple short and repeated sequences, and the length of which is generally 21 to 47 bp. Nine CRISPRs were involved in HJ-2, and length of repeated sequences ranged from 9 to 18 bp.

**Table 1:** General features of the genome of *P. peoriae* HJ-2

Feature	Value
Genome size (bp)	6,001,192
GC content (%)	45
Gene density	906.5genes/Mb
Genomic Islands	10
CDS	5237
Genes assigned to COG	3608(66.33%)
Genes assigned to KEGG	2423(46.27%)
rRNA	39
tRNA	108
CRISPR	9

**Fig. 2:**
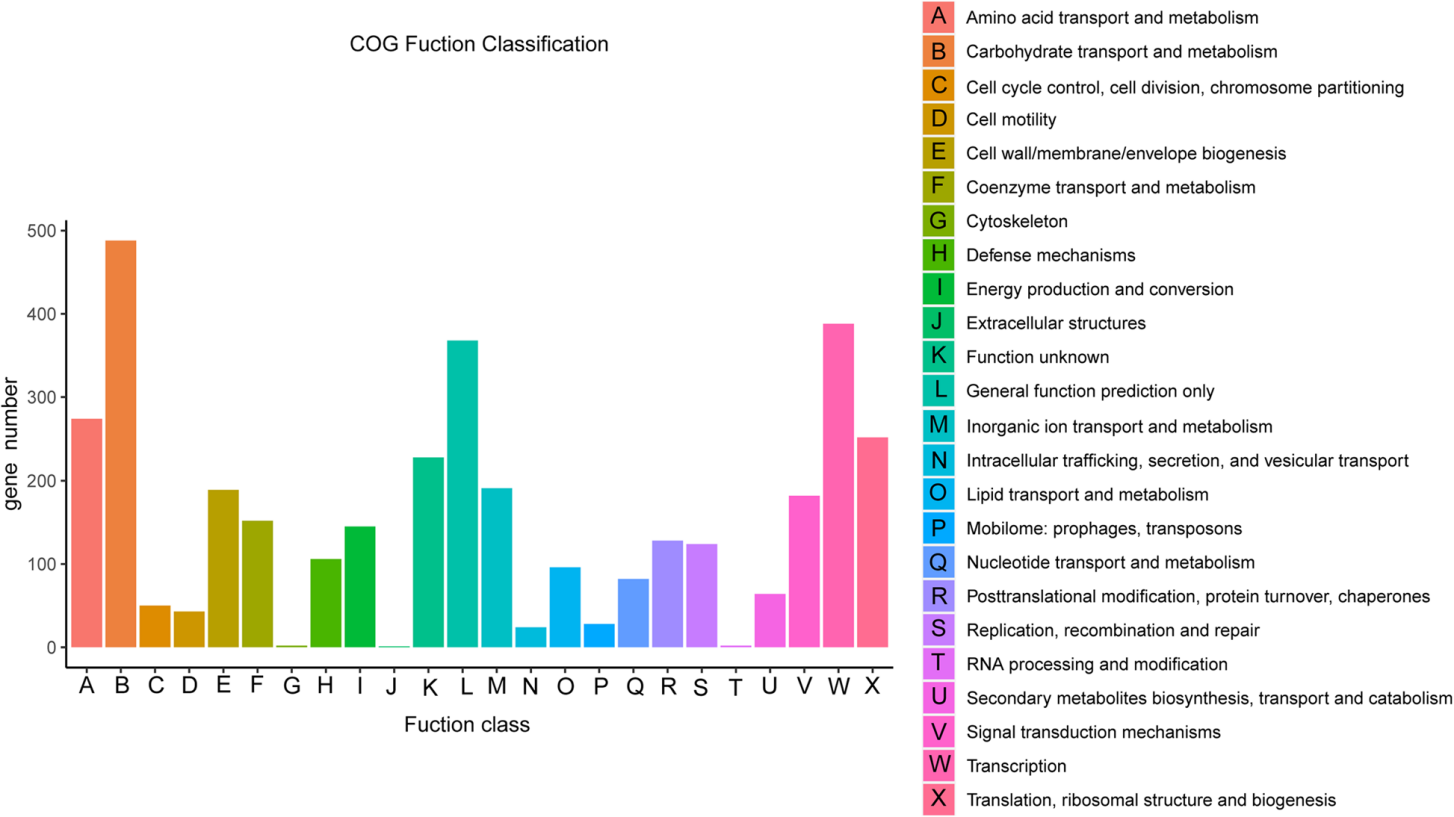
Distribution of genes across COG functional categories in the chromosome of *P. peoriae* HJ-2

According to GO annotation, a total of 2562 genes were classified into 27 functional groups, and the genes involved in biological process were most abundantly (Suppl. Fig.  1**)**. Among biological process group, the number of genes related to the metabolic process was highest, with 35.5% respectively. On the basis of COG database, a total of 3608 genes were assigned to 24 COG categories (Fig. 2). Carbohydrate transport and metabolism category represented the largest group (492 genes, 9.37% of all CDSs), followed by transcription, whereas only a small number genes were assigned to extracellular structures category. According to KEGG annotation, 2423 genes (46.27% of all CDSs) were assigned to 35 KEGG pathways, and the largest number of identified genes were classified into metabolism pathways. Among these pathways, the most represented pathways included carbohydrate metabolism(289 genes, 5.52% of all CDSs), followed by amino acid metabolism and energy metabolism pathways (Suppl. Fig. 2).

### Identification of strain HJ-2

HJ-2 was isolated from the rhizosphere of *P. polyphylla*, and cultured at 30 °C in Luria–Bertani broth. The 16S rDNA gene amplified from the genomic DNA of HJ-2 (approximately 1.4 kb) was sequenced (GenBank accession no. MK911741), and the BLAST search revealed that the sequence shared 99.72% identity to *Paenibacillus* spp*.*(e.g., *Paenibacillus peoriae* HS311, *Paenibacillus polmyxa* ATCC15970 and *Paenibacillus polmyxa* YC0573).

**Fig. 3:**
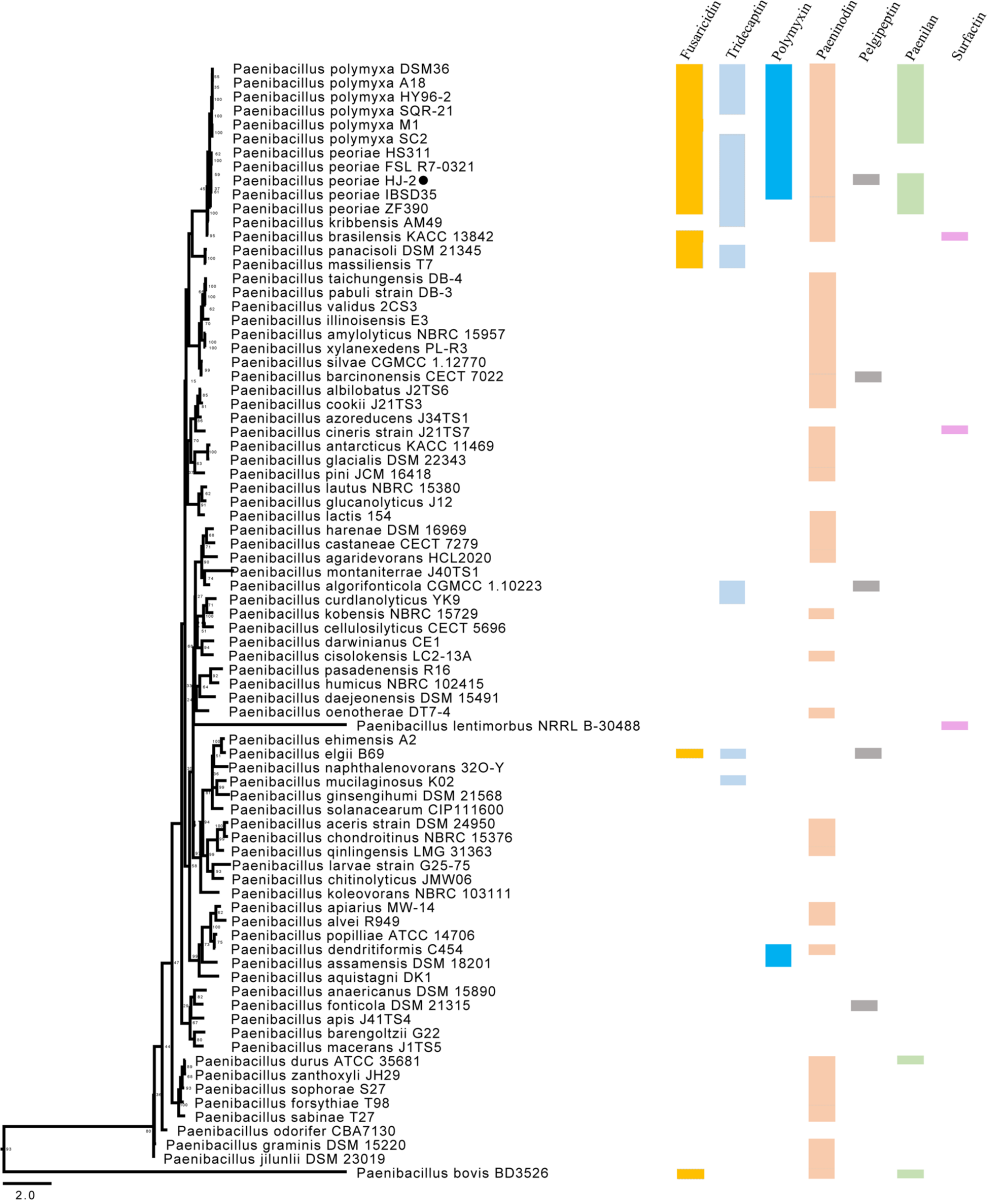
Phylogenetic tree for *P. peoriae* HJ-2 and the genus *Paenibacillus* based on homologous genes. Coloured blocks represent gene clusters for biosynthesis of fusaricidin, tridecaptin, polymyxin, pelgipeptin and surfactin detected in genus *Paenibacillus*, whilst white space represents gene clusters absence. Number in the branches represent bootstrap values. Scale bar represents sequence divergence

**Fig. 4:**
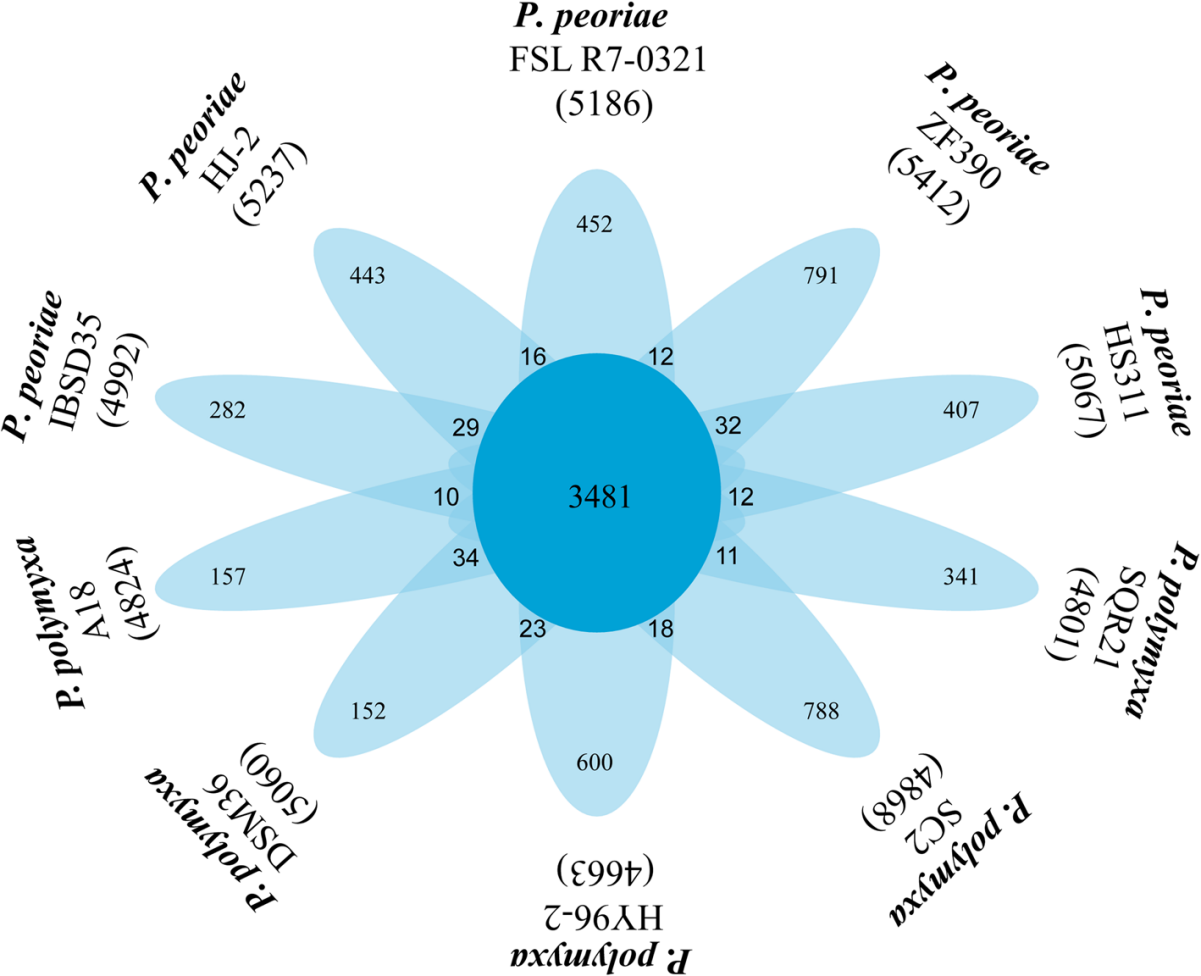
Flower plot representing the total (outermost layer), unique (second layer) (strain specific), and core proteins (center of the plot) in *P. peoriae* and other five *P. polymyxa* strains

Average nucleotide identity (ANI) is one of the most powerful approaches for evolutionary distance assessment between bacterial species based on digital whole genome comparison. Based on ANI values, the genome sequence of HJ-2 displayed highest similarity with the species of *P. peoriae* with the ANI values over 96%, whereas the ANI values between HJ-2 and other strains were lower, and ranged between 64 and 90% (Suppl. Fig. 3). By applying whole-genome analysis, the phylogenetic tree construction based on the single-copy genes from the 79 *Paenibacillus* genomes available in the Genbank database demonstrated that HJ-2 appeared to belong to *P. peoriae*, with the closest relative to *P. peoriae*IBSD35 (Fig. 3). We also performed a pangenome analysis to compare HJ-2 with other nine strains (*P. peoriae* HS311, IBSD35, FSLR7-0321, ZF390; *P. polymyxa* SQR21, SC2, HY96-2, DSM365, A18). As shown in Fig. 4, 3,481 orthologous protein coding genes are conserved and constitute the core genome. In addition, the number of gene families unique to strain ZF390 was 791, which was the highest among all of the analyzed strains. The annotation revealed that these specific genes encoded a large number of transcriptional regulators, helicase domain proteins, hypothetical proteins, aminotransferases, transposases, drug resistance transporters, chloramphenicol resistance proteins, etc. Nucleic acid co-linearity results showed that strain HJ-2 has high co-linearity with *P. peoriae* HS311 (Suppl. Fig. 4).

### Biocontrol potential of strain HJ-2

**Fig. 5:**
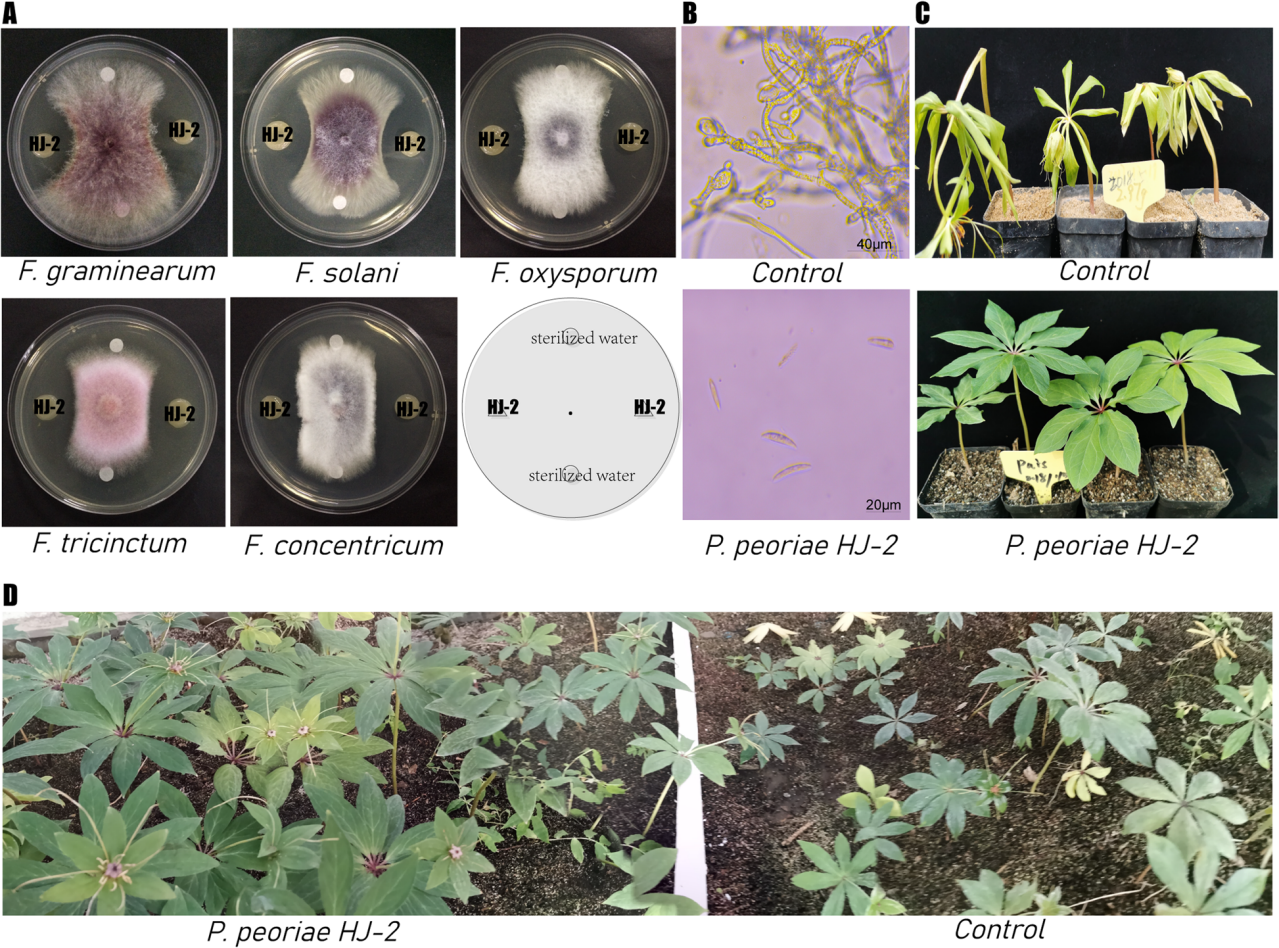
Strain HJ-2 could serve as a potential biocontrol agent against stem rot on *P. polyphylla* (**A**), antagonistic activity of strain HJ-2 against plant pathogens in vitro (**B**), effect of HJ-2 on spore germinations of *F concentricum* (**C**), biological control effect of HJ-2 against stem rot on *P. polyphylla* in the greenhouse (**D**), biological control effect of HJ-2 against stem rot on *P. polyphylla* in the field

As shown in Fig. 5A, the strain HJ-2 presented antagonistic activity against five *Fusarium* spp*.* in vitro. HJ-2 exerted maximum antifungal activity against *F. tricinctum* and *F. concentricum.* The antifungal activity of HJ-2 against *F. solani* and *F. graminearum* s.str. were lowest (Suppl. Table 2)*.* In addition, HJ-2 could inhibit the spores germination of *F. concentricum* (Fig. 5B)*.* Based on the above results, we infer that HJ-2 has the potential to suppress stem rot on *P. polyphylla*. To verify this hypothesis, we conducted greenhouse and field experiments, and the results indicated that HJ-2 could significantly control stem rot on *P. polyphylla* in both the greenhouse and the field. The symptoms of stem rot on *P. polyphylla* in the HJ-2 treatment were significantly weaker in compared with the control treatment (Fig. 5C and D). The incidence rate of stem rot on *P. polyphylla* with the HJ-2 treatment was 35.3% in greenhouse and 11% in the field, which was significantly lower than that (89.2%, 52%) with the control treatment (Table 2).

**Table 2:** Evaluation of *P. peoriae* HJ-2 on biocontrol efficacy and the plant growth parameters of *P. polyphylla* in greenhouse and field experiments

Parameter		*P. polyphylla*			
		**Greenhouse experiment**		**Field experiment**	
		**Control**	**Treatment**	**Control**	**Treatment**
Disease incidence (%)		89.2 ± 0.3a	35.3 ± 0.4c	52.0 ± 0.2b	11.0 ± 0.15d
Control efficacy (%)		53.9 ± 1.3		41.0 ± 0.6	
Stem	Length(cm)	5.6 ± 0.2c	8.8 ± 0.3b	7.6 ± 0.4bc	11.2 ± 0.6a
	Fresh weight(g)	12.3 ± 0.7c	15.8 ± 0.4bc	18.3 ± 0.5b	25.3 ± 0.7a
	Dry weight(g)	2.1 ± 0.1c	2.7 ± 0.2c	4.3 ± 0.1b	7.1 ± 0.3a
Root	Length(cm)	1.5 ± 0.1c	2.1 ± 0.2b	2.3 ± 0.2b	3.5 ± 0.3a
	Fresh weight(g)	4.5 ± 0.2c	6.7 ± 0.3bc	7.3 ± 0.2b	9.2 ± 0.4a
	Dry weight(g)	0.6 ± 0.1c	1.2 ± 0.1b	1.6 ± 0.2ab	2.7 ± 0.2a

The data are means ± SDs. The different lowercase letters in the same column indicate significant different at the *P* < 0.05 level, using LSD test

In addition, the plant growth promotion capability of HJ-2 was evaluated in the greenhouse and field experiments. As shown in Table 2, the growth parameters (the length, fresh and dry weight of stem and root) of *P. polyphylla* with the HJ-2 treatment were significantly higher than those of the control treatment. In the field experiment, HJ-2 exhibited a significant effect on plant growth-promoting in compared with the control treatment (Suppl. Fig. 5). The results of IAA production, nitrogen fixation, and phosphate solubilization assays indicated that the strain HJ-2 possessed most common PGP characteristics (Suppl. Fig. 6).

### Colonization and biofilm formation of strain HJ-2

**Fig. 6:**
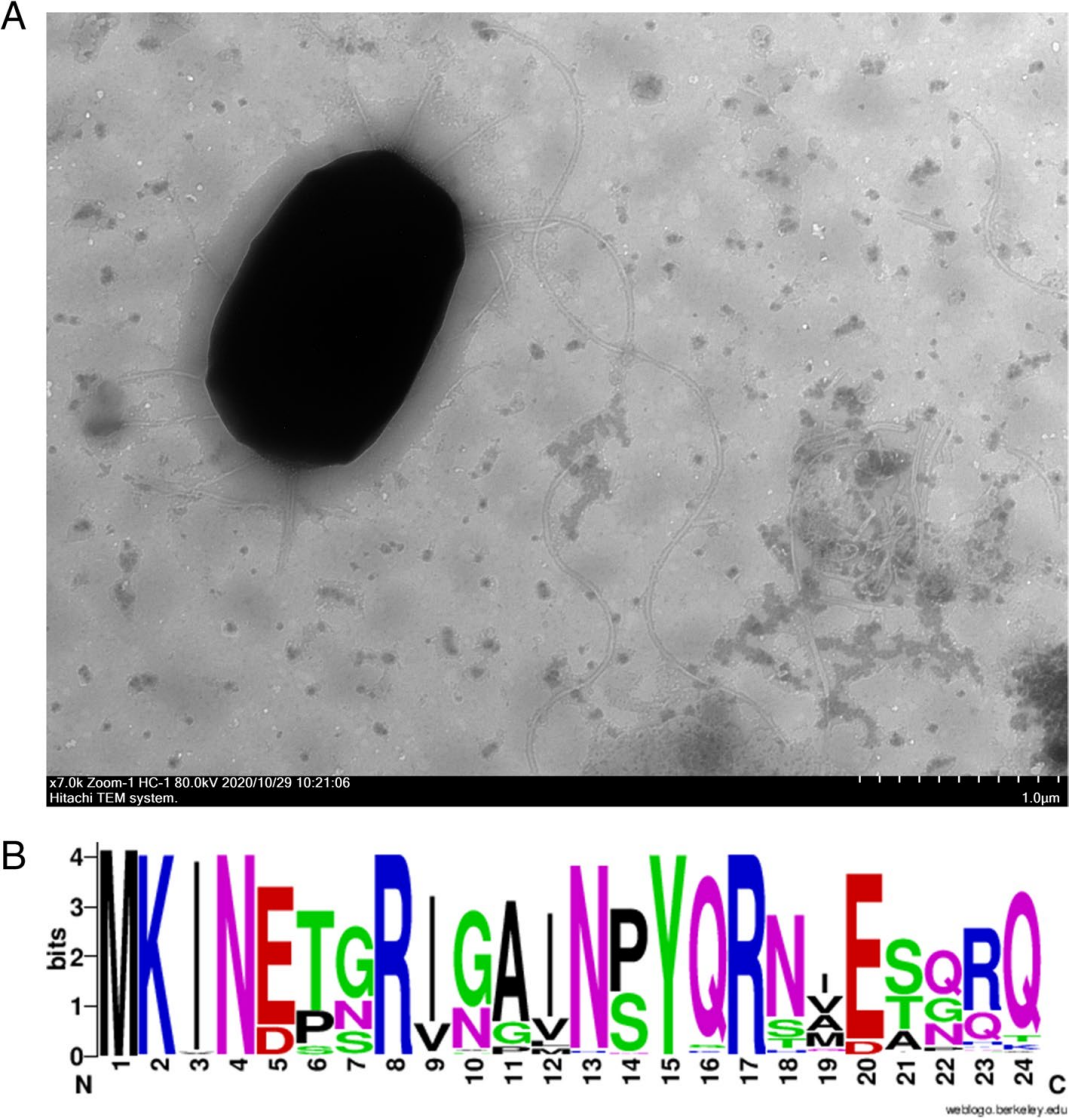
**A** Transmission electron microscopy section of HJ-2 (**B**), Conservation of flg22 motif. The N-terminal of FliC proteins of HJ-2 shown a highly conserved motif shared with *Paenibacilus* flg22

**Fig. 7:**
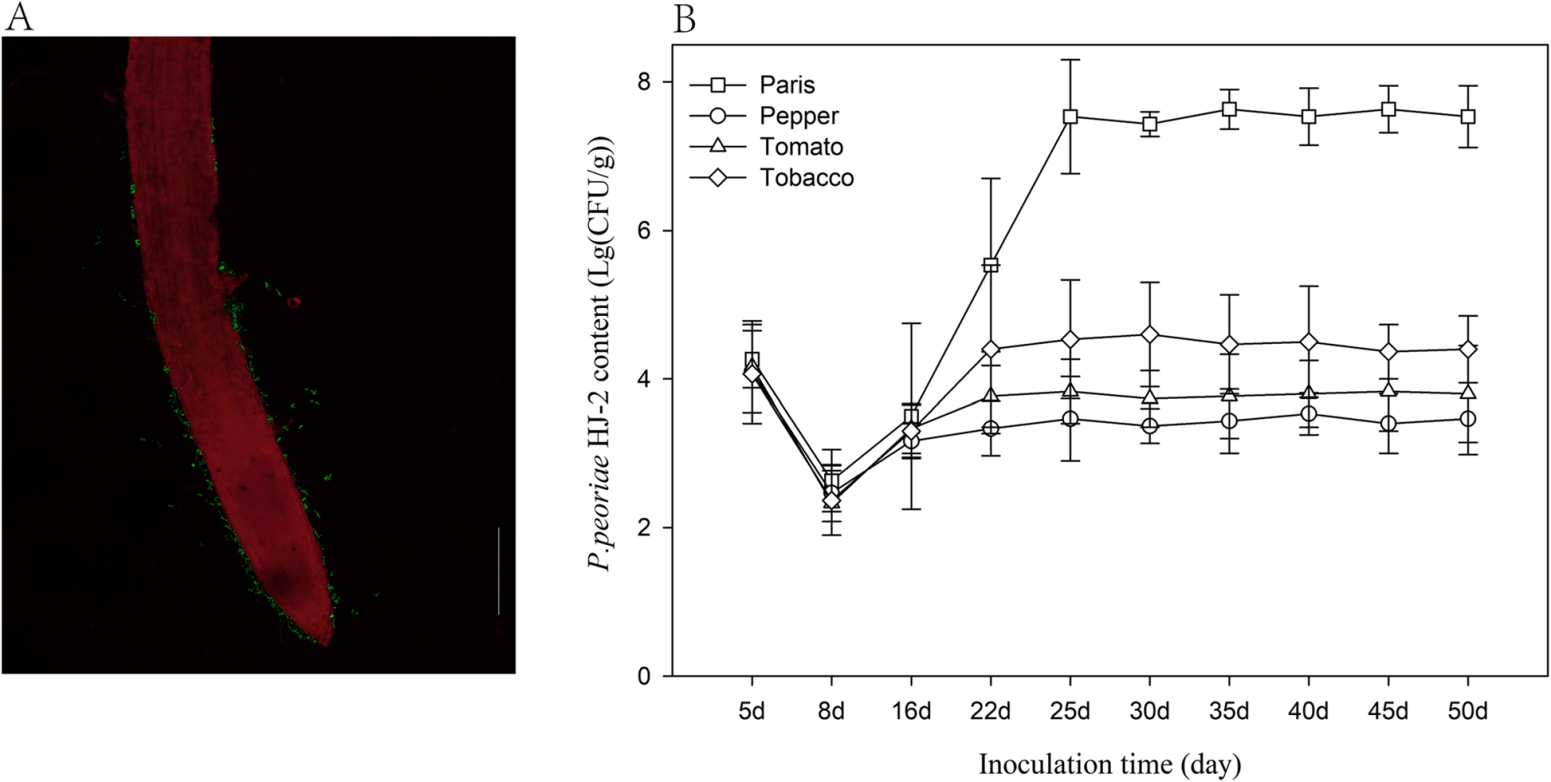
Colonization of P. peoriae HJ-2 on the seeding roots (**A**), Green fluorescence protein (GFP)-tagged *P. peoriae* HJ-2 mainly colonized the *P. polyphylla* roots (**B**), the content of effectively colonized bacteria on the roots of four plants. Bars = 100 µm

As shown in Fig. 6A, arrangements of flagella in HJ-2 is peritrichous, and multiple flagella arise from along the cell body. On the basis of previous researches, core genes involved in the assembly of flagellum, such as *flg*BCDEGKL, *fli*AEGHJLMPRSW, *flh*ABFG, m*otA* and *motB* were detected in genome of HJ-2 (Suppl. Table 3) [27]. Flagellin containing N-terminally conserved flg22 was also found in the *P. peoriae* HJ-2 (Fig. 6B). As shown in Fig. 7, bacterial cells attaching on the surface of roots could be observed after 16 days of inoculation. The number (3.16 ± 0.15 × 10^7^ CFU/g) of strain HJ-2 stably colonizing in the rhizosphere of *Paris* was superior to those in the other HJ-2-inoculated plants (pepper: 2.93 ± 0.2 × 10^3^ CFU/g; tomato: 6.3 ± 0.28 × 10^3^ CFU/g; tobacco: 2.5 ± 0.5 × 10^4^ CFU/g, respectively) after inoculation for fifty days. During the colonization process, the population of the strain declined dramatically during the next eight days after inoculation, and then began to increase until finally stable colonization.

The core genes involved in biofilm formation pathways were selected from KEGG database for comparison between the strain HJ-2, ZF390 and HS311 using BLAST. As shown in Table 3, key genes involved in biofilm formation were found in genome of HJ-2, ZF390 and HS311, and the sequence identity exceeded 97%. The sequence identity between HJ-2 and HS311 exhibited higher than that between strain HJ-2 and ZF390.

**Table 3:** Comparison of core genes involved in biofilm formation in strain HJ-2, HS311 and ZF390

Gene name	Location		Product	Identity(%)	
				**ZF390**	**HS311**
*kinB*	2,363,269	2,364,546	sensor kinase	97.03	97.73
*spoOF*	3,760,041	3,760,352	stage 0 sporulation protein F	98.72	99.04
*spoOA*	1,218,318	1,219,121	stage 0 sporulation protein A	97.76	98.88
*degU*	3,007,565	3,008,287	response regulator	98.2	99.45
*degS*	3,008,292	3,008,813	sensor histidine kinase	99.81	100.00
*AbrB*	3,635,054	3,635,596	transcriptional regulator	99.26	99.82
*spoOB*	2,363,269	2,364,546	sporulation sensor kinase	97.03	97.73
*rapZ*	3,796,330	3,797,229	RNase adapter protein	98.22	99.56

### Genes /gene clusters for antibiotic synthesis and induction of plant resistance

On the basis of antiSMASH database, twelve clusters related to secondary metabolite synthesis were identified in HJ-2. Among these gene clusters, three clusters were specific and existed only in HJ-2, while nine clusters existed in more than one strain (Suppl. Table 4). Six clusters involved in antifungal and antibacterial peptides (fusaricidin; polymyxin, tridecaptin, pelgipeptin, paenilan and paeninodin) biosynthesis were found in genome of strain HJ-2 (Table 4). However, no gene clusters encoding the biosynthesis of pelgipeptin or paenilan were detected in HS311, and the gene clusters encoding the biosynthesis of polymyxin or pelgipeptin were not detected in ZF390. According to the comparison of gene clusters involved in biosynthesis of fusaricidin and tridecaptin, the result shown that the two gene clusters sequences in strains HS311 and ZF390 exhibited very high similarities with those in strain HJ-2, with the similarity of 99.3%, 93.2% and 98.4%, 95.5%, respectively.

**Table 4:** Comparison of gene clusters involved in antibiotic biosynthesis in strain HJ-2, HS311 and ZF390

Antibiotic name	Activity	Location			Identity (%)	
		**HJ-2**	**HS311**	**ZF390**	**HS311**	**ZF390**
Fusaricidin	Broad antimicrobial activity against *Fusarium* sp., also suppresses G^+^ bacteria [28]	3,650,067 -3,719,981	63,180 - 131,651	62,571 - 131,051	99.3	98.4
Tridecaptin	Suppresses G^−^ bacteria [29]	89,772- 182,664	2,578,853 -2,671,371	2,419,321 -2,511,835	93.2	95.5
Polymyxin	Broad antimicrobial activity, especially against G^−^bacteria [30]	2,710,256 -2,790,093	5,116,022 - 5,197,037		95.1	
Pelgipeptin	Broad antimicrobial activity against G + and G^−^ bacteria [31]; PelgipeptinA and PelgipeptinB against *Fusarium graminearum* and *Rhizoctonia solani* [32]	485,090 - 558,941	-	-	-	-
Paenilan	Suppresses G^+^ bacteria [33]	5,331,079 - 5,358,085	-	1,620,879 - 1,647,885	-	96.2
Paeninodin	Broad antimicrobial activity against G + and G^−^ bacteria [34]	5,011,316 - 5,035,438	1,439,160 - 1,463,275	1,301,862 - 1,325,980	97.2	96.3

Based on numerous reported examples of elicitor, the genes coding for resistance inducers were selected for comparison between the strainHJ-2, ZF390 and HS311 using BLAST. As shown in Table 5, the genes coding for several elicitors, such as 2, 3-butanediol, acetoin, peptidoglycan and EF-Tu were all detected in HJ-2, ZF390 and HS311, meanwhile *flgL* was detected in the HJ-2 and HS311except for the strain ZF390. The sequence identity between HJ-2 and HS311 exhibited higher than those between strain HJ-2 and ZF390.

**Table 5:** Comparison of genes involved in synthesis of resistance inducers in strain HJ-2, HS311 and ZF390

Resistance inducers	Plant resistance type	Gene name	Location		Product	Identity(%)	
						**ZF390**	**HS311**
2,3-Butanediol	ISR	*alsS*	5,251,050	5,251,535	Acetolactate synthase	98.77	98.97
		*alsD*	5,947,927	5,948,673	Acetolactate decarboxylase	98.80	98.80
		*bdh*	1,893,383	1,893,679	2,3-butanediol dehydrogenase	96.26	97.98
Acetoin	ISR	*alsD*	5,947,927	5,948,673	Acetolactate decarboxylase	98.80	98.80
Peptidoglycan	PTI	*dacA*	3,710,123	3,710,332	carboxypeptidase	94.39	95.81
Flagellin	PTI	*flgL*	2,996,323	2,996,535	Flagellin	-	92.45
EF-Tu	PTI	*tuf*	2,113,547	2,113,711	elongation factor Tu	96.96	98.71

## Discussion

*Bacillus peoriae* was originally recognized as a new species of gas-producing *Bacillus polymyxa* on the basis of DNA relatedness, multilocus enzyme electrophoresis analysis, and other phenotypic characteristics. It was later reclassified as *Paenibacillus peoriae* with an emended description of the species. Phylogenetic reconstruction based on the single-copy genes from the nomenclatural type strains of currently recognized *Paenibacillus* species has clearly demonstrated that the species *P. peoriae* is closely related to *P.polymyxa*, and gene clusters involved in antifungal and antibacterial peptides (fusaricidin; polymyxin, tridecaptin, paenilan and paeninodin) biosynthesis have been found encoded in the genomes of *P. peoriae* and *P.polymyxa*. Pair-wise ANI values for the HJ-2 and five *P.polymyxa* strains ranged between 89.8 and 89.9%. Meanwhile, pair-wise ANI values for HJ-2 and four *P. peoriae* strains ranged between 96.5 and 97.3%, which were considerably higher than the above percentage range. Under the assumption that ANI values of 95–96% indicate bacterial species boundaries, these results are congruent with the phylogenetic tree.

Due to the advantages of plant growth promotion and broad-spectrum antimicrobial activity, the species *P. peoriae* is a potential BCA used as biofertilizer. However, limited number of comprehensive studies have revealed the biocontrol mechanism of *P. peoriae* to date. With the aim of providing some insight into biocontrol mechanisms in molecular level, the genome of *P. peoriae* HJ-2 was completely sequenced. In contrast to other species of *P. peoriae*, the genome of HJ-2 is smaller than that of HS311 (6,219,810 bp) and ZF390 (6,383,990 bp), and was found to share 3510 orthologous genes with IBSD35. This number is slightly larger than those (HJ-2 vs HS311, 3421; HJ-2 vs ZF390, 3436; HJ-2 vs FSL R7-0321, 3497) shared between HJ-2 and other three *P.peoriae* strains, reflecting the closer phylogenetic relationship of *P.peoriae* HJ-2 and *P.peoriae* IBSD35. Based on the results of genome assembly and annotation report, numerous coding genes for rRNAs were found in *P. peoriae* strains. The genome-encoded divergent rRNAs regulate gene expression at the ribosome level in bacteria.With the characteristic of possessing numerous rRNAs, soil microorganisms have capacities to rapidly cope with ceaseless nutritional compositions changes [35, 36]. GIs are composed of integrated foreign DNA fragments, which are frequently associated with pathogenesis, metabolism and antibiotic resistance [37]. GIs are important players in genome plasticity, thus supporting their rapid adaptation. Bacteria have multiple immune functions to remove exogenous virus genes by CRISPRs [38, 39]. The result suggests that the strain HJ-2 successfully resisted bacteriophages invasion. No plasmid has been identified when assembling the *P.peoriae* HJ-2 genome sequence data.

Effective colonization is a prerequisite for PGPRs to implement their biocontrol function. Colonization of PGPRs are influenced by various factors, such as root exudates and environmental factors. Plant could recruit beneficial rhizobacteria via secreting metabolite, and the major inducible root-secreted metabolite selectively activated chemotactic mobility of rhizobacteria [40]. Rhizobacteria finally reach the surface of plant roots by flagella-driven motion. However, a few PGPRs colonization is not limited to a specific region in the plant (such as rhizosphere), and they can be transported to other tissues using transmission systems (e.g. bacterial endophytes). In the process of colonization, bacterial endophytes often produces many enzymes, such as endoglucanases and endopolygalacturonidases [41]. To examine the ability of HJ-2 to colonize plant roots, we labeled the strain with GFP. In the present study, HJ-2-gfp cells were found to be attached to the surface of *Paris* roots. We also found that the bacterial cells could colonize on pepper, tomato and tobacco roots, but lesser than that on *Paris* roots. As a signal to attract or repel microbes, the root exudates serve as a carbon source for soil microorganisms. Therefore, we surmise that the root exudates of *Paris* contain one or more signaling molecules that directly bind to receptor domains. Such direct binding enables a highly sensitive response over a wide dynamic range of background ligand concentrations. The formation of biofilm is a dynamic process involving an attachment stage, accumulation stage, maturation stage and dispersal stage. The cells residing in the biofilm are encased within a self-produced exopolymeric matrix that commonly comprises lipids, proteins (frequently exhibiting amyloid-like properties), eDNA and exopolysaccharides [42]. This matrix fulfills a variety of functions for the community, from providing structural rigidity and protection from the external environment to controlling gene regulation and nutrient adsorption [43]. Previous studies have revealed the signaling pathway for biofilm formation in *B.subtilis*, the signals are sensed through histidine kinases(KinA-KinD) that phosphorylate Spo0F, Spo0F∼P transfers the phosphate to Spo0A, and Spo0A∼P leads to SinI accumulation and matrix gene expression [44]. Among this signaling pathway, Spo0A is a key transcription regulatory factor that controls the expression of genes involved in biofilm formation and sporulation [45]. Meanwhile, biofilm formation is negative regulated by Rap family of phosphatases, which lower the Spo0A∼P level in the cell, and prevent sporulation [46]. To date, the signaling pathway of biofilm formation has not been reported in *P. peoriae*. In this study, core genes involved in biofilm formation were detected in genome of HJ-2, ZF390 and HS311, with high sequence identity. Therefore, the signaling pathway for biofilm formation in *P. peoriae* probably possess high similarity with those reported in *B.subtilis*.

PGPRs have attracted considerable attention owing to their demonstrated ability to solubilize mineral phosphates, fix nitrogen, synthesize phytohormones and degrade lignocellulose and increase plant tolerance to abiotic stress by reducing host ethylene levels through 1-aminocyclopropane -1-carboxylate (ACC) deaminase activity [47]. In this study, greenhouse and field experiments have confirmed that selected strain HJ-2 could improve the growth of physical parameters in *P. polyphylla*. IAA plays a vital role in plant growth and development as a regulator of numerous biological processes. The capacity for IAA production of HJ-2 was proved in vitro by using LC/MS method. According to the KEGG database analysis, genes encoding key enzymes in the IAA biosynthesis were found in strain HJ-2 (Suppl. Table 5). Another strategy that PGPRs use to enhance plant growth is nitrogen fixation. *P. peoriae* HJ-2 established nitrogen-fixing potential through the ARA method. As reported in N_2_-fixing strains within the genus *Paenibacillus*, nitrogen fixation is carried out by molybdenum-dependent nitrogenases, which are encoded by a conserved *nif* gene cluster (comprised by nine genes: *nif*B, *nif*H, *nif*D, *nif*K, *nif*E, *nif*N, *nif*X, *hes*A, and *nif*V) [48]. According to the KEGG database analysis, six of these genes were also detected in genome of HJ-2 (Suppl. Table 5)**.** At present, the excessive use of nitrogen fertilizer leads to environment pollution. The detrimental effect may be lessened by using the nitrogen-fixing rhizobacteria, and *P. peoriae* HJ-2 could be utilized as biofertilization in agriculture.

The genus *Paenibacillus* is known for its potential to produce a series of bioactive compounds, including non-ribosomally synthesized lipopeptides (LPs), polyketides and ribosomally synthesized peptides [49]. LPs (e.g. polymyxins, pelgipeptin, surfactins, and fusaricidins) have been reported as strong antibacterial agents mostly active against phytopathogens [50, 51]. Fusaricidins displayed excellent antifungal activities against many plant pathogenic fungi, especially *Fusarium* spp, in vitro [52]. The antifungal mechanism of fusaricidin is through permeabilization and disruption of the cell membraneis. The production of fusaricidins by *P. polymyxa* is encoded on the NRPS gene cluster called *fus* with eight genes(*fusA*-*fusH*) [53]. In addition to *P. polymyxa*, we also found the *fus* cluster existed in species of *P. peoriae*, and the majority of these gene clusters are conserved in all *P. peoriae* strains. Polymyxins and tridecaptin have been described in species of *P. polymyxa for* possessing strong antimicrobial activity against Gram-negative bacteria. On the basis of antiSMASH database, the majority of these gene clusters were also detected in genomes of *P. peoriae* strains except for ZF390. Pelgipeptins were first discovered as secondary metabolites in *Paenibacillus elgii*, and the variants A and B display antifungal activity against several soil borne pathogens, including *Fusarium graminearum* and *Rhizoctonia solani*. The gene cluster encoding pelgipeptin biosynthesis was merely detected in genomes of *P. peoriae* strainsHJ-2, and was not typical in other *P. peoriae* strains. The diversifications of antibiotic gene clusters in *P. peoriae* presumably explain the differences of their target profiles and efficiency against phytopathogens.

In addition to producing a spectrum of antimicrobial peptides, *P. peoriae* HJ-2 produces antibacterial proteins, most of which are cell wall-degrading enzymes synthetized by ribosomes, such as β-1,3-glucanase and chitinase. β-1,3-glucanase can hydrolyze the cell wall of most plant-pathogenic fungi, thus inhibiting the growth of the hyphae. The β-1,3-glucan metabolism enzymes mainly include three important enzymes: endo-β-1,3-glucanase, exo-β-1,3-glucanase and β-1,3-glycosyltransferase [54]. According to the Carbohydrate-Active enZYmes Database, a series of endo-β-1,3-glucanases are produced by *P. peoriae* HS311. Based on the analysis of the KEGG database, genes encoding endoglucanase were also found in *P. peoriae* HJ-2 (Suppl. Table 6). β-1,3-glucanase produced by *Gliocladium catenulatum* inhibited *Fusarium* spp. growth, conidia germination and degraded the cell walls of the pathogen [55]. The inhibition of the spore germination and hyphal growth of pathogenic fungi by fusaricidin or β-1,3-glucanase or both is not well understood. Based on the current data and previous studies, the activities of β-1,3-glucanase are repressed by glucose and reduced under an acidic pH.

ISR can be triggered by PGPRs or fungi and lead to resistance priming against subsequent exposure to biotic and abiotic stresses. Several compounds secreted by PGPRs have been identified as bacterial elicitors responsible for ISR, such as 2, 3-butanediol, acetoin and surfactin [56]. The genes coding for several elicitors were detected in genome of HJ-2, ZF390 and HS311, with high sequence identity. Bacterial flagellin or EF-Tu is a general conserved elicitor that results in intracellular signaling in defense responses known as pathogen or microbe-triggered immunity (PTI/MTI) [57]. Flagellin containing N-terminally conserved flg22 was also found in *P. peoriae* HJ-2 (Fig. 6B). Previous studies have shown that the plant growth-promoting rhizobacteria could elicited reactive oxygen species (ROS) burst in plant leaves and roots, and PGPR tolerated higher oxidative stress than plant pathogen via two-component regulatory system ResDE. According to this mechanism, PGPR can successfully colonize in both root and leaf of plants [58]. To infer, *P. peoriae* HJ-2 could trigger ISR and accelerate defenses against plant pathogen.

## Conclusions

In summary, the results of this study indicate that *P. peoriae* HJ-2 could serve as a potential BCA against stem rot on *P. polyphylla*. The genome of HJ-2 consists of a single 6,001,192 bp chromosome with an average GC content of 45% and 5,237 predicted protein coding genes, 39 rRNAs and 108 tRNAs. The phylogenetic tree indicated that HJ-2 is most closely related to *P. peoriae* IBSD35. Based on genome analysis, the genome of HJ-2 contains more than 70 genes and 12 putative gene clusters related to secondary metabolites, which have previously been described as being involved in chemotaxis motility, biofilm formation, growth promotion, antifungal activity and resistance inducers biosynthesis. The underlying biocontrol mechanisms can be inferred as follows: (1) Plant recruits PGPR to colonize in the rhizosphere via secreting metabolite; (2) Biofilm formation and antibiotics biosynthesis protect plant against pathogen infection; (3) PGPR greatly revitalize plant growth through nitrogen fixing, phytohormones biosynthesis and phosphate solubilization; and (4) ISR can be triggered by PGPR and lead to resistance priming against biotic and abiotic stresses, etc. This study may provide a scientific basis for the further optimization of biofertilizers based on *P. peoriae* HJ-2 in terms of field application. The knowledge obtained can be further translated into comprehensive strategies for establishing sustainable agricultural practices by using biocontrol agents to suppress plant pathogens.

## Materials and methods

### Isolation of rhizosphere bacteria

Soil samples (50 g) were collected from *Paris* *polyphylla* roots in a herb plantation of Saiwudang, Shiyan, Hubei Province, China (32°27′58″N; 110°40′45″E). Bacteria was isolated with the dilution plating method. Subsamples (5 g) were diluted with 50 mL of sterile distilled water, thoroughly dispersed by shaking (150 r/min) for 30 min at 28 °C, and further diluted 10^3^–10^7−^fold. A 100 μL of the diluted samples was spread onto Luria–Bertani (LB) agar plates and maintained at 25 °C for 24 h. After incubation, the bacterial colonies were picked and repeatedly restreaked onto agar plates until their purity was confirmed for 16S rRNA gene analysis. The isolated strains were maintained at -80 °C in LB media with glycerol (30%, v/v) for long-term storage.

### In vitro antagonism test

To evaluate the biocontrol potential of *P. peoriae* HJ-2, we performed a co-cultivation assay on PDA medium in vitro. Five *Fusarium* spp*.* including *F. oxysporum*, *F. graminearum* sensu stricto, *F. solani* var*. coeruleum* (Sacc.) Booth., *F. concentricum* and *F. tricinctum* were used as pathogenic fungus. *F. oxysporum and F. concentricum* which had been reported causing stem rot on *P. polyphylla* in China were isolated in our lab from infected *P. polyphylla*. The 6 mm plugs from the edge of pathogenic fungus were inoculated in the center of PDA medium (90 mm in diameter), and then the HJ-2 was inoculated on both sides of the culture dish by using sterile paper disks (8 mm in diameter), filter paper with sterile water was used as the control. After incubated for 7 days at 25 °C, the colony diameters were measured and recorded. Effect of *P. peoriae* HJ-2 on *F. Concentricum* spore germination assays were performed as described by Jiang [59]. The top surface of *P. peoriae* HJ-2 cultured in LB broth was sliced and removed. Subsequently, the sublayer was transferred to a 1.5 mL centrifuge tube (sterile). The centrifuge tube was inoculated with 10 μL conidium suspension of *F. concentricum* (1 × 10^5^ conidia/mL), and incubated for 24 h at 25 °C. The germination of conidia was observed using Phenix BMC500 microscope (Phenix China, Inc.). The experiment was conducted three times with two replicates per treatment.

### Biocontrol experiments in greenhouse and field

To evaluate plant growth promotion and biocontrol effect of *P. peoriae* HJ-2, greenhouse and field experiments were carried out in this study. For the greenhouse experiment, the seeds of *P. polyphylla* were sown into autoclaved soil with one seedling per pot and then cultivated in a greenhouse at 20/25 °C (night/day) with 70% humidity and 14-h photoperiod. The seedling was treated with 10 mL of bacterial suspension of HJ-2 at OD_600_ of 0.8 by sprinkling the root in combination with spraying the leaf when the seedling grew to six leaves, and sterile water served as a control. Ten days later, the seedling in each treatment was inoculated with 10 mL spore suspensions of *F. concentricum* (1 × 10^5^ conidia/mL). The length, fresh weight, dry weight of roots and stems, and the incidence rate of disease were recorded and photographed after inoculation for twenty-five days. All experiments were conducted three times with twenty seedlings per treatment.

Moreover, the field experiments were conducted in a herb plantation of Saiwudang, Shiyan, Hubei Province, China (32°27′58″N; 110°40′45″E), and the experiments did not involve endangered or protected species. Two treatments were established, and water was used as a mock control. A 20 mL of bacterial suspension of HJ-2 at OD_600_ of 0.8 was poured onto the roots, and sprayed on both sides of the leaves when the seedlings grew to six leaves. Fifty days later, the effects on plant growth promotion and the incidence rate of disease were recorded and photographed. All experiments were conducted three times with fifty seedlings per treatment.

### Indole 3 acetic acid (IAA) production, nitrogen fixation, and phosphate solubilization assays

The production of IAA was measured using LC/MS method previously described [60], with some modifications. A 20 μL of bacterial suspension at OD_600_ of 0.5 (10^6^—10^7^ CFU/mL) was added to 20 mL of liquid Landy medium (20 g/L glucose, 5 g/L glutamic acid, 1 g/L KH_2_PO_4_, 0.5 g/L MgSO4·7H_2_O, 0.5 g/L KCl, 5 mg/L MnSO_4_, 0.16 mg/L CuSO_4_, 0.15 mg/L FeSO_4_, 2 mg/L L-pheny- lalanine, 1 g/L yeast powder). The medium was maintained at 28 °C for 72 h by shaking (160 r/min), and the culture was centrifuged at 4 °C with 8000 rpm for 2 min. Then, a 100 mL of filtrate was collected with a 0.22 μm microporous membrane, and extracted with ethyl acetate for three times. The organic solvents were collected and dissolved with methanol after vacuum drying for LC–MS analysis. An Agilent 1100 Series LC/MS system and an Agilent Zorbax Exteng-C18 chromatographic column (2.1 mm × 150 mm, 3.5 μM) were used. IAA (Sigma) were prepared by methanol dissolution, and each standard sample had a concentration of 5 × 10^−7^ g/L.

Nitrogen fixation ability of HJ-2 was tested using the acetylene reduction assay (ARA), as described by Boddey [61]. A 20 μL bacterial suspension at OD_600_ of 0.5 was inoculated to 4 ml of semi-solid (0.18% agar–agar) NFb media. After incubation for 72 h at 28 °C in the dark, 10% (v/v) of the air phase was replaced with acetylene. The amount of C_2_H_4_ was measured using a gas chromatograph (Agilent 7890A) after incubation for 1 h with acetylene. The protein concentration of bacteria was collected and determined by using protein extraction kit (TaKaRa, DaLian, China).

The ability of phosphate solubilization was tested as previously described [62]. A 5 μL bacterial suspension at OD_600_ of 0.5 was inoculated to NBRIP medium (0.5% Ca_3_(PO_4_)_2_, 1% glucose, 0.01% (NH_4_)_2_SO_4_, 0.5% MgCl_2_, 0.02% KCl, 0.025% MgSO_4_·7H_2_O, 1.5% agar). After incubation for ten days at 28 °C, the growth of bacterial was recorded. The experiments were conducted three times.

### Colonization assays with the strain HJ-2 on seedling roots

The GFP-labelled *P. peoriae* HJ-2 was constructed with the pHT01EGFP plasmid, which carried the *gfp* and *Cm*^*R*^ genes. The competent cells of HJ-2 and transformation were obtained as described previously [63]. The seeds of four plants (*P. polyphylla*, pepper, tomato and tobacco) were surface-sterilized by soaking in 20% sodium hypochlorite solution for 20 min and cultured in flowerpot with autoclaved soil. When the roots of seeding were approximately 2 cm (cm) in length, a 10 mL of bacterial suspension at OD_600_ of 0.8 was inoculated onto the roots. For GFP observation, root surfaces were rinsed with sterile water and stained with 10 μg ml^−1^ propidium iodide (PI) for 15 min. Excitation and emission wavelengths for detecting the GFP- tagged HJ-2 were 488 and 510 nm, respectively. Excitation and emission wavelengths for detecting the PI- stained root were 535 and 617 nm, respectively. The colonization of the strain HJ-2 on seedling roots was observed using NikonDS-Ri2 microscope (Nikon Japan, Inc.).

Bacteria counting was performed by using the plate counting method with LB medium containing Chloramphenicol (Cm, 5 μg mL^−1^) as described previously [64]. The roots of four plants were harvested after inoculation for 5 d, 8 d, 16 d, 22 d, 25 d, 30 d, 35 d, 40 d, 45 d and 50 d, and washed twice with phosphate buffer (1 M, pH 7.0). Then, the effectively colonized bacteria was remove from roots to sterile water. Last, the CFU count was recorded after 48 h of incubation at 28 °C, and the sterile water was applied as control. All bioassays and experiments were conducted three times with twenty seedlings per treatment.

### DNA extraction, PCR amplification, 16S rRNA gene analysis

Genomic DNA was extracted with a DNA Mini Bacteria Kit (Invitrogen, Shanghai) following the manufacturer’s instructions. The 16SF-(AGAGTTTGATCCTGGCTCAG) and 16SR-(GGTTACCT- TGTTACGACTT) universal primers were used for PCR amplification [65]. The 16S rRNA gene was sequenced by Life Technologies Inc. (Shanghai, China) and manually aligned with reference sequences retrieved from the GenBank database following BLAST searches for fast identification.

### Whole-genome sequencing and annotation

DNA was extracted from cells harvested from LB broth culture medium of HJ-2 with a Genomic DNA extraction kit (TaKaRa, DaLian, China). The whole genome was sequenced using the PacBio Sequel platform. Reads were assembled using HGAP (version 2.3.0, SMRT Analysis) [66]. The assembly data for the complete genome have been deposited in GenBank with the accession number PRJNA580302. Coding DNA sequence (CDS) prediction was performed using Glimmer 3.02 [67]. A circular map of the genome was obtained using Circos version 0.64 [68]. Genomic islands (Gis) were predicted using the IslandPath- DIOMB GI prediction method [69]. tRNAs and rRNAs were predicted using tRNAscan-Sev1.3.1 and Barrnap 0.7 software4, respectively [70]. Clustered regularly interspaced short palindromic repeat sequences (CRISPRs) were identified using MinCED [71]. Functional annotation was based on BLASTP searches (BLAST 2.2.28 +) against the NCBI nonredundant (NR) database and gene database, the STRING database, and the Gene Ontology (GO) database. Based on the string database, BLASTP comparisons were used to perform Clusters of Orthologous Groups of proteins (COG) annotation, according to which protein functions could be classified [72]. The BLAST algorithm was used to compare the predicted genes with the KEGG database, and the corresponding genes involved in specific biological pathways were identified according to the KEGG Orthology (KO) numbers obtained from the alignment [73]. GO was annotated with Blast2GO [74].

### Genome comparison

103 genome sequences of *Paenibacillus* spp*.* were obtained from GenBank. The accession numbers of the strains used for the analysis are provided in Supplementary Table S1. Phylogenetic Tree was conducted by using the Phylogenetic Tree Building Service available at the Patric website (https://www.patricbrc.org), with codon tree method and 1000 genes selected for analysis as option [75]. ANI values were computed by using OrthoANI Tool version 0.93.1. Heatmap of the ANI matrix was computed using Morpheus (https://software.broadinstitute.org/morpheus) with Hierarchical clustering applied using euclidian distance matric and complete linkage clustering method. Pangenome analysis was conducted for *P. peoriae* HJ-2 and other nine strains by using OrthoMCL software [76]. Nucleic acid co-linearity was assessed for *P. peoriae* HJ-2 and *P. peoriae* HS311 by using MUMmer 3.0 software [77]. The gene clusters for secondary metabolites (containing antibiotics) in *P. peoriae* HJ-2 were annotated using the antiSMASH database version 4.0.2, and the other antibiotics were selected based on previous studies [78]. BLAST was used to compare the identities of the genes or gene clusters between HJ-2 and other strains.

### Transmission electron microscopy (TEM) section of HJ-2

A single colony from the LB agar plate was inoculated into 20 mL of liquid medium. After incubation, the medium was maintained at 30 °C for 12 h by shaking (160 r/min). A bacterial suspension at OD_600_ of 0.5 was gathered and washed with phosphate-buffered saline (PBS) (pH = 7.2). Then, the strain was negatively stained with 2% phosphotungstic acid (Sigma). Finally, the stained bacteria was deposited on a carbon-coated grid, followed by observation under a HT-7700 transmission electron microscope (HT-7700, Hitachi High-Tech Corporation, Tokyo, Japan).

### Statistical analysis

All datas were analysed by using analysis of varianceinSPSS24.0 (IBMSPSS Inc.,United States). Significant differences between means were compared by using the LSD test (Fisher’s protected least significant differences test) at *P* = 0.05. A *P* value < 0.05 was considered significant.

## Supplementary Information

**Additional file 1: Table 1.** Genome sequences used for analysis in this study. **Table 2.** The antifungal activity of HJ-2 against *Fusarium* spp. **Table 3.** Chemotaxis and assembly of flagella. **Table 4.** Secondary metabolite clusters identified in this study by using antiSMASH database. **Table 5.** IAA biosynthesis and nitrogen fixation. **Table 6.** The genes cording for endoglucanase in the *P. peoriae* HJ-2.

**Additional file 2: Figure 1.** GO annotation. **Figure 2.** Kyoto Encyclopedia of Genes and Genomes (KEGG) Pathway annotation. **Figure 3.** ANI values matrix heatmap. **Figure 4.** Nucleic acid co-linearity of strain HJ-2 with *P. peoriae* HS311. **Figure 5.** The growth-promoting effect of *P. peoriae* HJ-2 on *P. polyphylla*. **Figure 6.** IAA production, nitrogen fixation, and phosphate solubilization of HJ-2.

## Data Availability

The data reported in this paper have been deposited in the NCBI Sequence Read Archive (SRA) database (https://www.ncbi.nlm.nih.gov/subs/sra) under accession no. PRJNA580302.

## Abbreviations

TCM: Traditional Chinese medicine
PGPR: Plant growth-promoting rhizobacteria
JA: Jasmonic acid
ET: Ethylene
BCA: Biocontrol agent
IAA: Indole 3 acetic acid
GI: Genomic Island
GO: Gene ontology
COG: Clusters of Orthologous Groups
KEGG: Kyoto Encyclopedia of Genes and Genomes
ISR: Induced systemic resistance
TEM: Transmission Electron Microscopy
