# Supplementary material for: Complete genome sequence of biocontrol strain Paenibacillus peoriae HJ-2 and further analysis of its biocontrol mechanism
Source: BMC Genomics. 2022 Feb 24;23:161. doi: 10.1186/s12864-022-08330-0 (PMC8876185; doi:10.1186/s12864-022-08330-0)
Supplement: Supplementary file 2 — Additional file 2: Figure 1. GO annotation. Figure 2. Kyoto Encyclopedia of Genes and Genomes (KEGG) Pathway annotation. Figure 3. ANI values matrix heatmap. Figure 4. Nucleic acid co-linearity of strain HJ-2 with P. peoriae HS311. Figure 5. The growth-promoting effect of P. peoriae HJ-2 on P. polyphylla. Figure 6. IAA production, nitrogen fixation, and phosphate solubilization of HJ-2. [file 12864_2022_8330_MOESM2_ESM.doc]

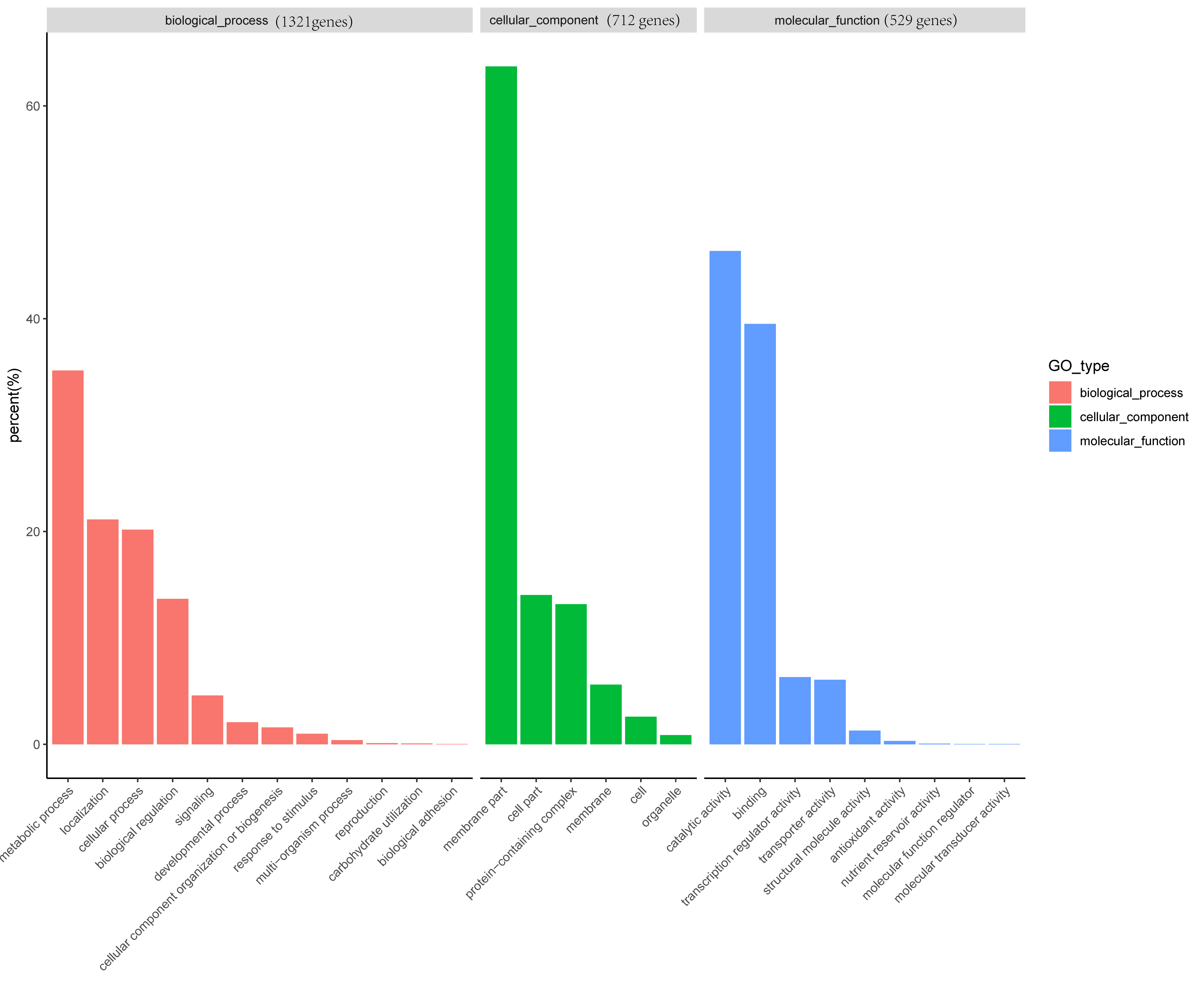


**Suppl. Figure 1. GO annotation**

Note: A total of 2562 genes were classified into 27 functional groups, and the genes involved in biological process were most abundantly.

`
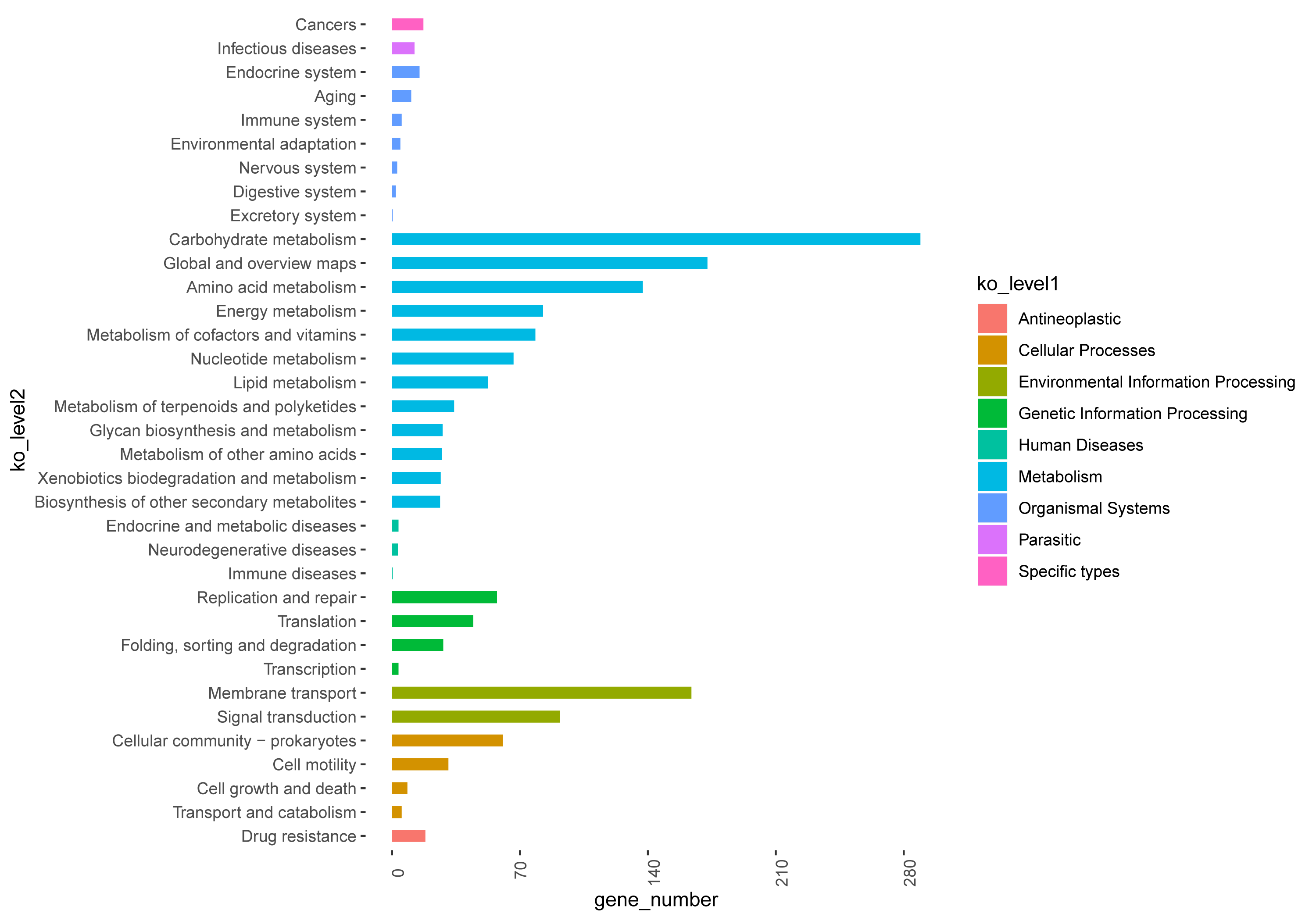


**Suppl. Figure 2. Kyoto Encyclopedia of Genes and Genomes (KEGG) Pathway annotation**

Note: 2423 genes (46.27% of all CDSs) were assigned to 35 KEGG pathways, and the largest number of identified genes were classified into metabolism pathways.

**Suppl. Figure 3. ANI values matrix heatmap**

Note : ANI values between HJ-2 and *P. peoriae* exceeded 96%.


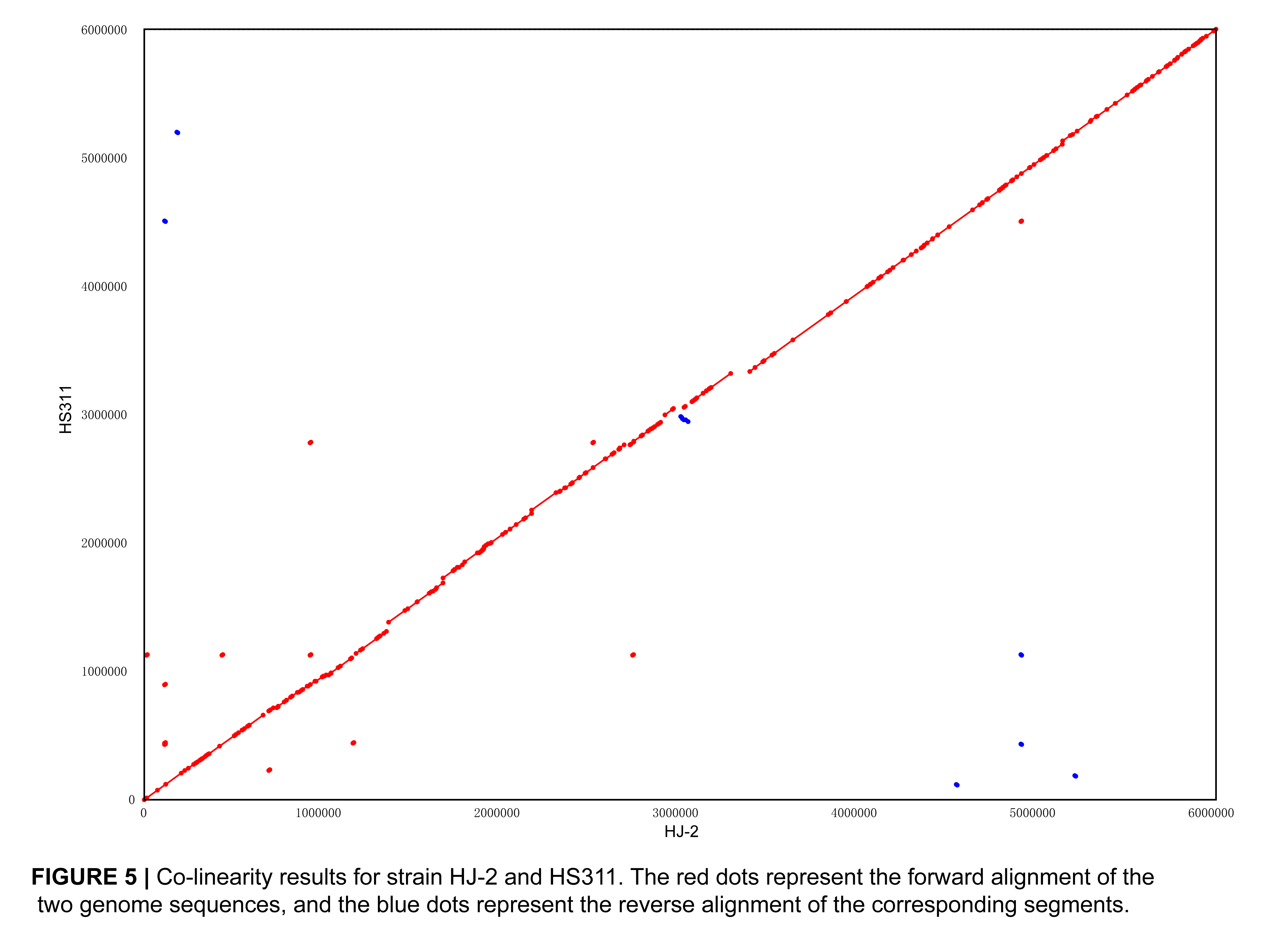


**Suppl. Figure 4. Nucleic acid co-linearity of strain HJ-2 with *P. peoriae* HS311**


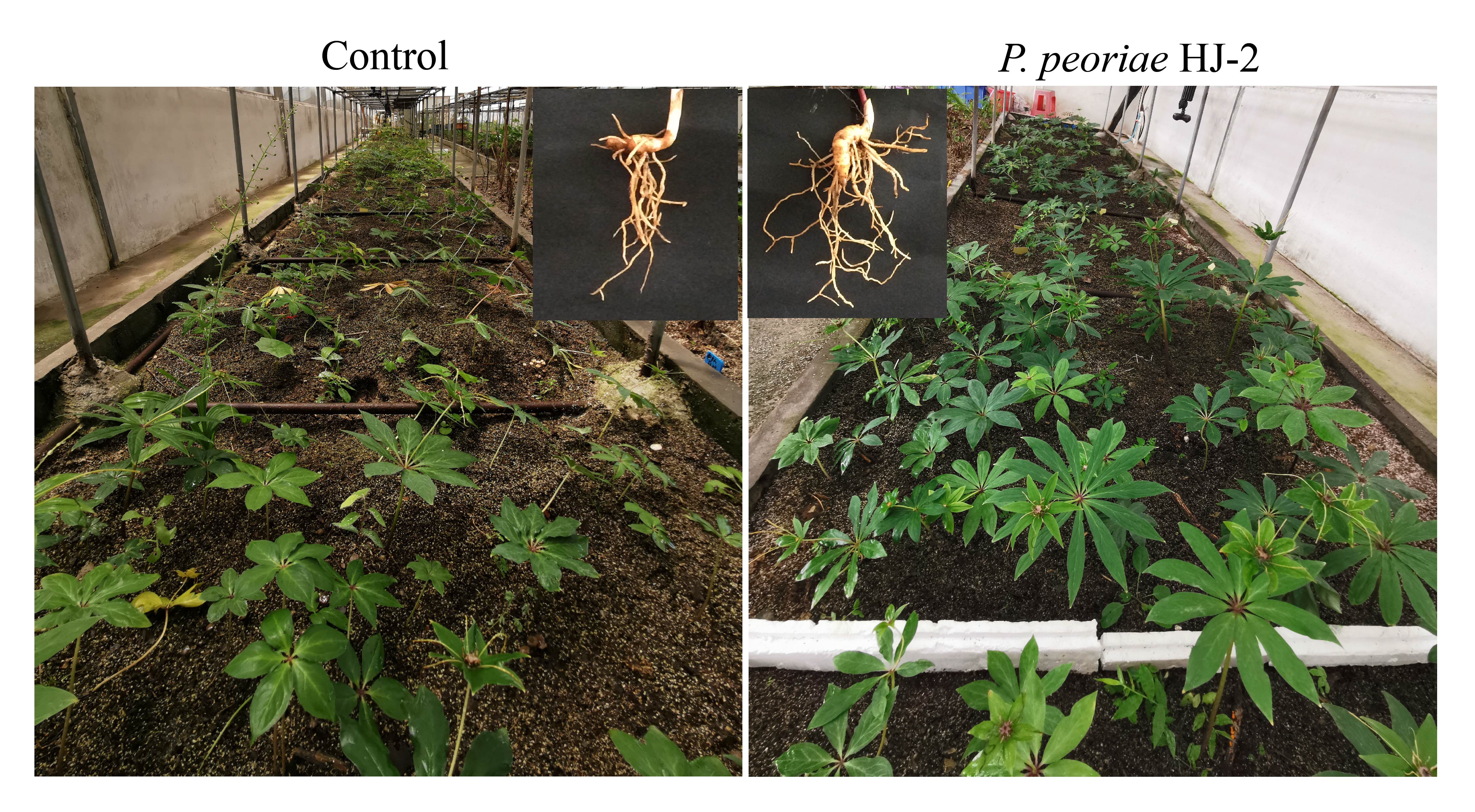


**Suppl. Figure 5. The growth-promoting effect of** ***P. peoriae* HJ-2 on *P. polyphylla*.**

Note: The Effects of beneficial rhizobacterium *P. peoriae* HJ-2 on the growth promotion to *P. polyphylla* in field experiments respectively.


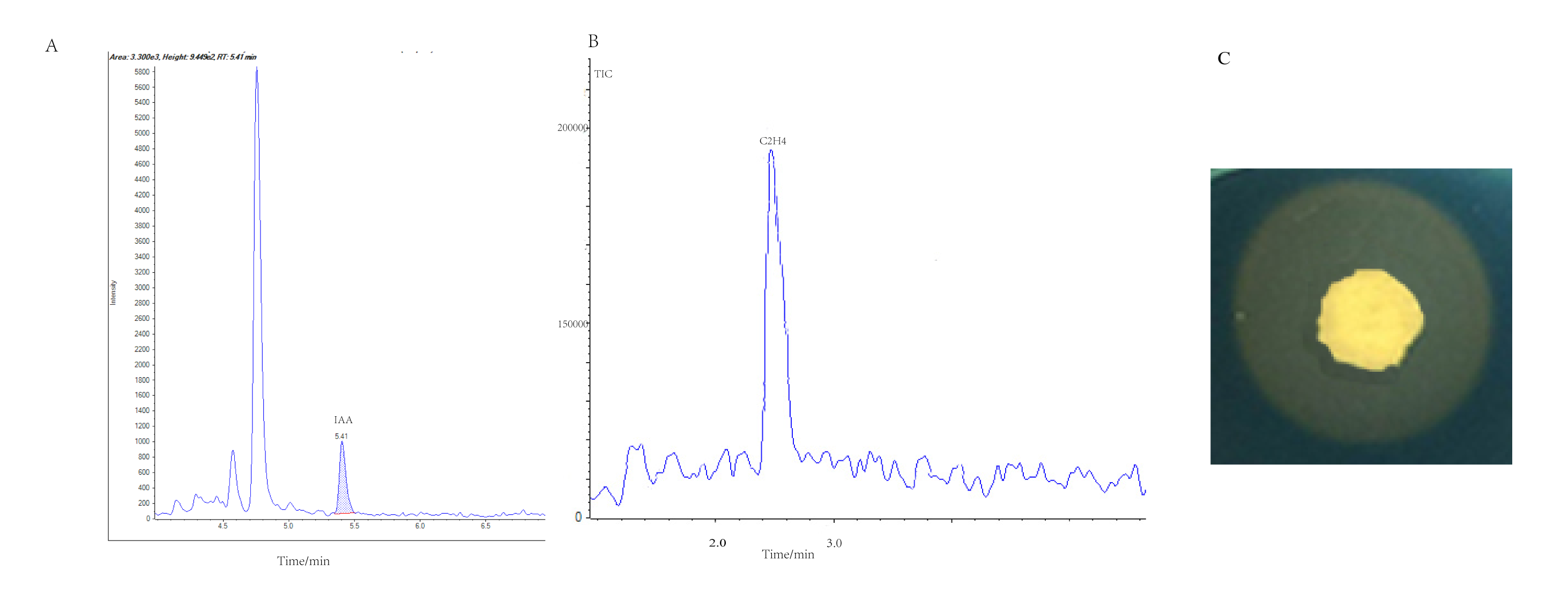


**Suppl. Figure 6.** IAA production, nitrogen fixation, and phosphate solubilization of HJ-2

Note: A: IAA production of HJ-2; B: nitrogen fixation of HJ-2; C: phosphate solubilization of HJ-2.

IAA :132.43mg/L

ARA (nmol C_2_H_4_ mg protein min^−1^ ) : 60.21
